# Effect of music on short-term pain perception during peripheral intravenous cannulation

**DOI:** 10.3389/fpain.2026.1923943

**Published:** 2026-10-09

**Authors:** Andrei Cristinel Dragnea, Lars Michels, Till Fellner, Argyro Christou, Giovanna Brandi, Giulio Bicciatto, Riccardo Ludovichetti, Thomas Frauenfelder, Zsolt Kulcsar, Meritxell Garcia Alzamora

**Affiliations:** 1 Department of Neuroradiology, University Hospital Zürich, Zürich, Switzerland; 2 Institute of Diagnostic and Interventional Radiology, University Hospital Zürich, Zürich, Switzerland; 3 Faculty of Medicine, University of Zürich, Zürich, Switzerland; 4 Experimental Pharmacopsychology and Psychological Addiction Research, Department of Psychiatry, Psychotherapy, and Psychosomatics, University Hospital of Psychiatry Zürich, Zürich, Switzerland; 5 Department of Music, Zürcher Hochschule der Künste (ZHdK), Zürich, Switzerland; 6 Institute for Intensive Care, University Hospital Zürich, Zürich, Switzerland; 7 Clinical Neuroscience Center, University of Zürich, Zürich, Switzerland

**Keywords:** intravenous cannulation, Mozart effect, music, pain perception, short-term pain

## Abstract

**Background:**

There is no general consensus on which music genre is most effective for a specific condition. The impact of music on subjective pain perception during intravenous cannulation for a magnetic resonance imaging (MRI) scan was investigated.

**Methods:**

204 patients were assigned to one of four groups: listening to Mozart, favorite music, relaxation music, or no music (control). Pain was assessed using the Visual Analogue Scale at four time points.

**Results:**

Favorite music significantly reduced pain perception both 30 s and 2 min after puncture while Mozart showed a trend towards reduced pain perception 2 min after puncture. When including only patients with baseline pain (*n* = 71), a significant pain reduction was also observed in the Mozart group 2 min, with borderline significance 30 s after puncture, whereas the favorite music group not only intensified reduced pain perception 30 s and 2 min but also caused significant pain reduction immediately after puncture. No effect on pain was found for the relaxation music and control groups.

**Conclusions:**

Music may be effective for pain relief in unpleasant settings involving short painful interventions. Favorite music should be considered first, whereby patients with baseline pain could also benefit from listening to Mozart, indicating a potential “Mozart effect” in this subgroup. These exploratory results need to be validated in future studies including a higher number of patients.

## 1 Introduction

Music is known to have a positive effect on mood, emotions, anxiety, and stress (1–5), and has been shown to be an effective non-pharmacological adjunct for several diseases and conditions, including in intensive care unit (ICU) patients (1, 4, 6–10), stroke (11), epilepsy (12–20), spatio-temporal reasoning (21), cognition and depression (22–26), and pain (7, 27–32).

Studies available on music in diseases have been performed with different music genres, including favorite music, different classical music composers, heavy metal, especially self-composed music, and live music (4, 8, 21, 26, 27, 33). However, there is no consensus on which type of music is most effective for a specific target. Verrucio et al. observed in electroencephalograms an increase of alpha band and background alpha rhythm activity (pattern linked to memory, cognition, and problem solving) in patients with mild cognitive impairment after listening to Mozart's Sonata KV 448 (26). Bodner et al. described an increase in spatio-temporal reasoning after listening to Mozart but not to Beethoven's “Für Elise” (21). Pastor et al. noted that different types of music induced heterogeneous responses in the brain in ICU patients indicating the possibility of using different types of music for different targeted brain responses (8). In a study across countries, Becker et al. reported that most respondents preferred to listen to music when experiencing pain with favorite music being the preferred music genre, followed by a smaller trend selecting classical music. Still, notable differences between cultural background and social groups were noted (30).

It is not astonishing that many studies continue to include Mozart's music since the so-called “Mozart-effect”, introduced in 1993 by Rauscher et al., has become so popular (34). There, an improvement in spatial task performance after listening to the first movement of Mozart's Sonata KV 448 in healthy subjects was described, lasting up to 15 min after the testing period. Even if this very first study on Mozart was limited by low statistical power and bias-related measurement artefacts (34), the Mozart effect has been a topic of constant debate, with various studies showing inconsistent results (35). Some authors reject a specific effect of music in general, including Mozart, on medical conditions (36). Nevertheless, the musical aspects of the peculiarity of Mozart's Sonata KV 448 (structural components, rhythm, tempo, long-term periodicity, etc.) have been studied, and beneficial effects of this sonata in health-related matters based on these findings have been proposed (16, 20, 37, 38).

Most studies testing the effect of music on pain have been performed in patients with chronic pain due to a medical condition, e.g., in musculoskeletal or rheumatology diseases (28, 31, 39). Regarding externally applied pain via vascular cannulation, a pain reduction effect immediately after cannulation while listening to Mozart's Sonata KV 448 has been shown in hemodialysis patients reporting pain and exposed to repeated cannulations in short time intervals (37). Two studies in neonates illustrated the positive effect of listening to Mozart lullaby music for pain reduction during heel prick (29, 32). The aim of this study was to analyze whether music, including Mozart, can modulate pain sensation in adults during venous puncture over a time period of two minutes immediately prior to a Magnetic Resonance Imaging (MRI) examination.

## 2 Materials and methods

### 2.1 Patient recruitment

The study, which was reviewed and approved by the local Ethical Committee (Cantonal Ethical Commission Zurich, Basec Number of the project: 2024-01391) was conducted from October 2024 to June 2025. All experiments were performed in accordance with relevant named guidelines and regulations. Informed consent was obtained from all study participants.

Altogether, 223 patients were recruited from the daily clinical practice including only outpatients scheduled for an elective (non-emergency) MRI of the brain, spine, or head & neck for a neurological disease (Table 1) requiring contrast and able to sign the informed consent. Considering 19 dropouts either for technical or patient-related reasons, 204 patients (age range 17–83 years, 107 females, 97males) completed the study and were included in the final analysis.

**Table 1:** Number of the types of MRI scans performed and pathologies/findings of the 204 analyzed patients.

MRI	Pathologies/Findings
156 Brain/Cranium	87 Neoplastic
13 H&N	43 Vascular lesions and tumors
25 Spine	24 Infectious/inflammatory/autoimmune
6 TMJ	4 Trauma
4 Brain and Spine	8 Degenerative
	3 Congenital
	16 Other
	5 Lesion of unknown origin, unclear
	11 Unspecific, or no or probable no clinical relevance, incidental finding
	16 No findings

H&N, head and neck; TMJ, temporomandibular joint. Please note that the number of pathologies slightly exceeds the total number of patients included as some patients had more than one pathology.

Patient recruitment, study information, and consent obtainment were conducted by two physicians (one senior neuroradiologist and one resident). First, patients were asked whether they would mind being exposed to music listening in the course of the study. If they agreed, study recruitment process was initiated. After her inclusion, we noticed that one female patient was 17 years old at the time of the study. She was contacted during the analysis, by which time she had turned 18, and gave written consent for her previously collected data to be used.

Exclusion criteria included moderate to severe hearing problems, medication influencing the sense of hearing, cognitive, psychiatric or psychological disorders, and illicit drug abuse with the last drug administration within the previous 18 h. In the case of baseline pain (*n* = 71), patients with position-dependent pain were excluded from the study (e.g., pain relief from sitting to supine position).

For assessment of the patients' study eligibility criteria, all patients had to complete two questionnaires. A short general and medical questionnaire for the assessment of the patients' general characteristics and condition (weight, height, handedness, past and present medical history, chronic pain, medication, illicit drug consumption, hearing difficulties), and the Hospital Anxiety and Depression Scale questionnaire (40).

Patients were asked to complete a musical background questionnaire, which also included the indication of one favorite music work or song, which was played in the course of the study in case of having been assigned to the favorite music group (Table 2). The total time for completion of all three questionnaires was less than 10 min.

**Table 2:** Selected favorite music works by patients assigned to group 2 (listening to favorite music).

Patient	Selected favorite music	Music genre(s)
1	Mozart—Lacrimosa	Classical
2	Mozart—K525 I	Classical
3	IMA—Petit Frère	French Hip-Hop
4	Laura Pausini—Strani amori	Pop
5	Robert Miles—Children	Dance/Electronic
6	Crash Test Dummies—Mmm	*Folk-Rock*
7	Fred again—Delilah	Dance/Electronic, UK R&B
8	Yuvan Shankar Raja—Chinna Chinna Kangal	Indian Pop
9	Zerb × Coldplay—feelslikeimfallinginlove	Alternative Pop/Rock
10	Bushido—Alles wird gut	Gangsta-Rap
11	Oliver Dragojević—Kad mi dođeš ti	Pop, Chanson, Klapa, Jazz, Blue-eyed Soul
12	Vivaldi—Le quattro stagioni	Classical (Baroque)
13	Robert Miles—Children	Dance/Electronic
14	Bastille—Pompeii	Indie-Pop/Rock
15	Sailor—Girls Girls Girls	Pop/Rock
16	In Flames—Come Clarity	Metal/Progressive Metal
17	AC/DC—Safe in New York City	Hard Rock, Classic Rock
18	Daler Mehndi—Tunak Tunak Tun	Indian Pop
19	Coro Valsella—La Montanara	Folk
20	Tricky—Nothing matters	Trip hop
21	Neelix, Ghost Rider, The Gardener & The Tree—Wonders	Trance
22	Adele—Someone Like You	Pop and Soul
23	Adele someone like you	Pop and Soul
24	Bilderbuch—Maschin	Pop Rock, Hip-Hop
25	Tyla—Push 2 Start	Amapiano, Pop, Contemporary R&B
26	Phil Collins—Another Day In Paradise	Pop, Rock
27	Alessandra Amoroso—Fino a qui	Pop
28	Lionel Richie—Hello	Pop
29	Susan Cadogan—Hurt So Good	Reggae
30	Beethoven—9th Symphony “Freude schöner Götterfunken”	Classical
31	Dire Straits—Brothers in Arms	Pop, Rock
32	Liricas Analas—Siemis	Spoken word, Hip-Hop, Rap
33	Robbie Williams—Angels	Soft Rock, Alternative/Indie, Seasonal
34	Rihanna-Umbrella	R&B/Soul, Rock
35	Beethoven- 5th Symphony, 2nd movement	Classical
36	Tina Turner (all, chosen music piece not documented)	R&B/Soul
37	Michael Jackson—Bad	Pop, Dance-Pop
38	Hecht—Heicho	German Pop
39	Romeo Santo—Eres mía	Bachata
40	Schubert—Ständchen	Classical
41	Ashmaze—Q	Japanese Rock
42	Beethoven—5th Symphony	Classical
43	Michael Jackson—Smooth Criminal	Pop, Dance-Pop
44	Dave Brubeck Quartet—Take Five	Jazz, Cool Jazz, Bebop, Smooth Jazz
45	Schwyzerörgeliquartett Stockhorn (all, chosen music piece not documented)	Swiss Folk Music
46	AC/DC—Hells Bells	Hard Rock, Classic Rock
47	Hecht—Fiji	French Indie, German PoP
48	Smetana—“Die Moldau”	Classical (Romantic)
49	Lea—Ich mag dich	German Pop
50	Tool—Pneuma	Progressive/Alternative Metal, Hardrock, Progressive Rock, Artrock
51	Elton John/Sacrifice	Rock

During patient recruitment and informed consent, patients were introduced to the Visual Analogue Scale (VAS) (41) for pain assessment using a schematic and color-coded version of the scale ranging from 0 (corresponding to “feeling no pain at all”) to 10 (corresponding to “worst pain in my life”). The short general and medical questionnaire included a question asking for the current pain level (first/baseline VAS assessment). Patients were told that in the case of the presence of baseline pain, the pain had to be persisting for a minimum of 48 h in either a localized or a general form, otherwise they were not included in the study.

### 2.2 Interventions and pain assessment

Recruited patients were assigned to one of four possible study groups (groups 1–4) according to the chronological order of the scheduled MRI examinations (patient 1-music group 1, patient 2-music group 2, patient 3-music group 3, patient 4-music group 4, patient 5-music group 1, patient 6-music group 2, etc.). As the study was conducted within the clinical routine which required highest flexibility (core team had to be present on that day, not all patients scheduled could be included due to various incidents, e.g., due to late appearance of patients, unexpected urgencies requiring changing the MRI schedule, etc.), this allocation system turned out to be the simplest and most uncomplicated one for a straightforward and well-arranged music allocation for participants keeping in mind that this approach rather represented a prospective non-randomized controlled study (pseudo-randomization, not a true randomization).

Groups were defined as follows: Listening to the first part of the first movement of Mozart's Sonata KV 448, interpreted by Alfred Brendel and Walter Klien (group 1, *n* = 52, 23 right-handed, 5 with chronic pain); listening to favorite music (group 2, *n* = 51, 22 right-handed, 3 with chronic pain); listening to relaxation music [selected music piece: “Source of the I Am/Quelle des Ich Bin” from Peter Uwe Piotter (https://www.youtube.com/watch?v = pRYX5m92LZw), group 3, *n* = 50, 30 right-handed, 0 with chronic pain]; and no music exposition serving as a control (group 4, control, *n* = 51, 29 right-handed, 3 with chronic pain). For patients in group 2 who named one music group or singer as favorite music but could not specify a respective work or song, the latter was chosen by one of the study participants. For groups 1–3 the music was already playing when the patients entered the MR room and lasted at least shortly after the last VAS assessment. After this time point (not part of the study anymore) the same music or another music (selected by the patients) continued to be played if it was wished by the patients. The music was played from outside (desktop computer) via speakers freely into the MR room.

Pain perception via VAS assessment occurred 4 times in total with the first evaluation during the questionnaires’ completion before venous access puncture approximately 3–10 min before entering the MR room.

Once the patient was positioned on the MRI table, an intravenous cannula was inserted by one of five trained technicians who participated in the study. The needle size (3 different cannulas) was selected according to the MRI protocol or predetermined by the patient's vascular condition. The puncture site depended on the patient's characteristics and availability with the antecubital fossa being the standard puncture site. Patients were asked for their VAS scale pain level immediately after (2nd VAS assessment) as well as approximately 30 s (3rd VAS assessment), and 2 min (4th VAS assessment) after venous puncture, considering these being adequate time points for pain evaluation in our study (29, 32, 37).

The three post-puncture VAS ratings were collected verbally by the same technician who performed the cannulation and immediately documented by her/him after having left the MRI room. No patient received any premedication (e.g., sedatives, tranquilizers or relaxation medication) prior to the MRI examination.

As patients were sitting during the 1st VAS assessment, in contrast to the 2nd to 4th VAS interrogations, patients who indicated baseline pain being position-dependent in the first baseline VAS evaluation (VAS > 1) were excluded at this time point.

All patients in the music groups (groups 1–3) were asked if they could hear the music well when being positioned on the MRI table. Music volume was standardized being equivalent to pleasant background sound, whereas the actual volume was louder as patients had earplugs inserted with adjacent foam pillows within the head coil on the MR table. As the venous puncture and VAS assessments took place before image acquisition, the superimposed inherent MR noise was neglectable.

### 2.3 Statistical analysis

Statistical analysis was performed with the software SPSS version 29.0 (SPSS, Inc., Chicago, IL). The VAS pain scores before puncture were normalized to baseline, as patients indicating “no pain” at baseline (VAS 0 in the first VAS assessment) could not indicate negative pain scores in the subsequent VAS assessments, i.e., if one patient reported no baseline pain (VAS = 0 at the time of first VAS-assessment) no positive effect by music could be obviously documented as the VAS does not include negative scores). This effect was considered in the statistical analysis. Two repeated-measures ANOVA with time (immediately after as well as 30 s and 2 min after puncture) as within-subject factor as well as with group as between-subject factor were performed. ANOVA1 included all patients independently of the presence of baseline pain (VAS ≥ “0” at baseline, *n* = 204) whereas ANOVA2 included only patients with baseline pain (VAS was > “0”, *n* = 71). In case of a significant (*p* < 0.05) main or interaction effect, *post-hoc* Tukey HSD (Honestly Significant Difference) tests were performed as comparative analyses, adjusting for multiple comparisons (false positive rate was kept at alpha level of *p* < 0.05). In addition, a repeated-measure ANCOVA was performed across all patients with the factor time and the covariates sex, age, and body mass index (BMI), (for raw data see Table 3), as well as with the covariates pathologies/MRI findings and chronic pain.

**Table 3:** Repeated-measure ANCOVA for between-group differences across all patients for the covariates weight, height, BMI, age, and sex.

Covariate	Mean	SD	Min	Max	Number (f/m)
Whole group					
Weight	75.53	16.36	39.00	134.00	
Height	172.05	9.36	145.00	193.00	
BMI	25.41	4.63	13.82	42.72	
Age	50.46	15.30	17	83	
Sex					107/97
Group 1					
Weight	75.92	18.23	40.00	120.00	
Height	172.56	10.09	145.00	193.00	
BMI	25.35	5.09	15.63	36.61	
Age	49.82	16.40	21	83	
Sex					26/26
Group 2					
Weight	73.61	17.60	44.00	134.00	
Height	172.22	10.18	150.00	191.00	
BMI	24.60	4.25	16.60	39.58	
Age	47.96	16.2	20	82	
Sex					25/26
Group 3					
Weight	77.22	14.15	51.00	110.00	
Height	171.08	9.03	153.00	190.00	
BMI	26.38	4.43	18.07	38.83	
Age	51.06	14.8	17	77	
Sex					28/22
Group 4					
Weight	75.41	15.31	39.00	122.00	
Height	172.31	8.19	158.00	188.00	
BMI	25.34	4.66	13.82	42.72	
Age	53.04	13.62	26	82	
Sex					28/23

A sample size of a minimum of 200 patients was calculated to be sufficient for a statistical power to detect clinically meaningful differences in pain outcomes between the four groups (42).

## 3 Results

### 3.1 Patients’ characteristics

There was no complication for any of the 204 patients completing the study in all interventions. All patients attributed to one of the three music groups confirmed to be able to hear the music in a comfortable volume when entering the MRI room and positioned on the MRI table.

From the 204 patients, 124 indicated to play an instrument.

The majority of the intravenous cannulations occurred in the elbow (*n* = 196), with the others at the back of the hand (*n* = 6), in the forearm (*n* = 1), and in the thigh (*n* = 1). Except in one patient who was punctured twice, in all other patients successful venous puncture access occurred at the first attempt. Most venous cannulations (159 from 204) were performed by two out of the five included technicians).

Three types of intravenous cannulas of slightly different sizes were used whereby the smallest cannula of 22 gauge (G) was most often used [deep blue, outer diameter (OD) 0.85 mm, length 25 mm, *n* = 164], followed by the 20 G cannula (pink, OD 1 mm, length 33 mm, *n* = 35) and the 18 G cannula (green, OD 1.3 mm, length 45 mm, *n* = 5).

From the 204 patients, 71 patients indicated to have baseline pain (VAS score > 0). The subgroup of patients with baseline pain showed following group distribution: 15 patients group 1 (Mozart), 20 patients group 2 (favorite music), 17 patients group 3 (relaxation music), and 19 patients group 4 (no music, control).

### 3.2 Continuous and categorial variables, and pathologies/MRI findings

No between-group differences were observed for the continuous variables weight, height, BMI, and age (*F* = 0.42, *p* = 0.74; *F* = 0.25, *p* = 0.86; *F* = 1.26, *p* = 0.29; and *F* = 1.0, *p* = 0.40, respectively) or the categorical variables sex, handedness, chronic pain, and treating technician (*p* = 0.86*; p* = 0.95; *p* = 0.82*;* and *p* = 0.34, respectively, Chi-Square tests). For the pathologies/MRI findings, there was only a group difference for vascular pathologies between group 1 (Mozart) and 3 (favorite music) (*p* = 0.0008).

When analyzing the patients’ BMI, the respective technician performing the venous puncture, and the type of venous cannula used as independent variables, no influence on pain perception by any of these three variables was observed for patients with or without baseline pain >0 (all *p*'s > 0.28). A subgroup analysis including only patients who indicated a classical music work as favorite music piece was not possible due to the low number of patients fulfilling this criterion (*n* = 26).

### 3.3 Groups’ comparisons: VAS

A main effect of time was seen for both ANOVA's [*F*_(2)_ = 49.3, *p* < 0.001 and *F*_(2)_ = 20.1, *p* < 0.001, respectively] and ANCOVA [*F*_(2)_ = 5.73, *p* = 0.004]. Thus, comparative analyses were performed between all possible pairs of groups (six in total) for both analyses, once including all patients (with and without baseline pain at initial VAS assessment, ANOVA 1) as well as with baseline pain patients only (ANOVA 2). The results are summarized in Table 4 and Figure 1 (ANOVA 1) as well as in Table 5 and Figure 2 (ANOVA 2), respectively. The favorite music group showed significantly lower VAS scores compared to the control group (no music, group 4) both 30 s and 2 min post puncture both in the analysis including all patients (ANOVA 1; *p* = 0.03 and *p* = 0.009) and in the analysis with baseline pain patients (ANOVA 2; *p* = 0.015 and *p* = 0.006). Lower VAS scores immediately after puncture were only observed for the favorite music group in the subgroup analysis including baseline pain patients (ANOVA2, *p* = 0.025). The ANCOVA confirmed the results seen for ANOVA1, i.e., lower VAS scores in group 2 (favorite music) compared to group 4 (no music) at 30 s post puncture and 2 min post puncture (*p*-values as reported above).

**Table 4:** *Post hoc p*-values from time-dependent comparison of group analysis including all patients (with and without baseline pain (ANOVA 1).

ANOVA 1 (including all patients, with and without baseline pain, *p < 0.05)				
Group comparison	music types/no music	Immediately post puncture	30 s post punctrue	2 min post puncture
Mozart	Favorite music	0.618	0.522	0.672
Mozart	Relaxation music	0.866	1	0.952
Mozart	No music	0.864	0.493	0.179
Favorite music	Relaxation music	0.199	0.520	0.354
Favorite music	No music	0.194	0.030*	0.009*
Relaxation	No music	1	0.512	0.451

* *p* < 0.05.

**Figure 1:**
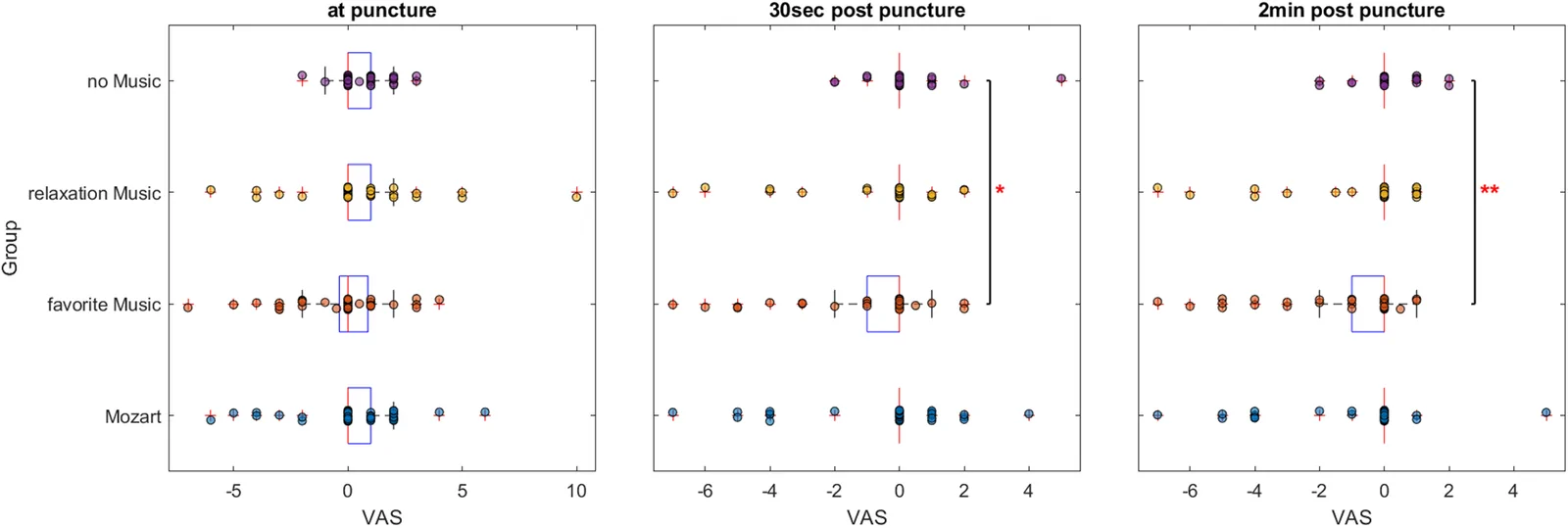
Box-plots of VAS-changes including all 204 patients (with and without baseline pain, VAS ≥ “0”). VAS scores were lower (*p < 0.05 and **p < 0.01, Tukey HSD tests) for the group opposition favorite music (group 2) vs. no music (group 4) 30 s post puncture and 2 min post puncture. Black line: median (50th percentile), blue line: interquartile range (25%–75%), dashed line: Whiskers (1.5* interquartile range).

**Table 5:** *Post hoc p*-values from time-dependent comparison of group analysis including including only patients with baseline pain (ANOVA 2).

ANOVA 2 (including only patients with baseline pain, *p < 0.05)				
Group comparison	music types/no music	Immediately post puncture	30 s post punctrue	2 min post puncture
Mozart	Favorite music	0.802	0.994	1
Mozart	Relaxation music	0.999	0.855	0.569
Mozart	No music	0.286	0.054	0.018*
Favorite music	Relaxation music	0.711	0.674	0.443
Favorite music	No music	0.025*	0.015*	0.006*
Relaxation	No music	0.318	0.261	0.299

* *p* < 0.05

**Figure 2:**
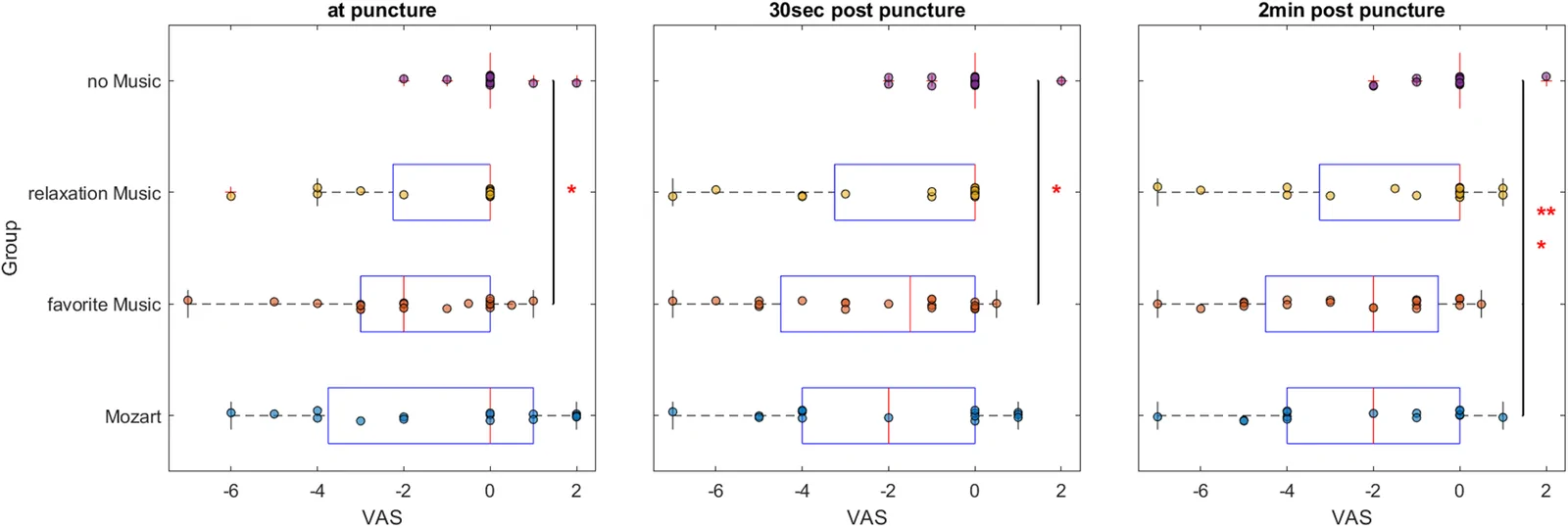
Box-plots of VAS-changes including only patients with baseline pain, VAS > “0” (*n* = 71). VAS scores were lower (*p < 0.05 and **p < 0.01, Tukey HSD tests) for the group opposition favorite music (group 2) vs. no music (group 4) at puncture, 30 s post puncture, and 2 min post puncture. In addition, Mozart (group 1) showed lower VAS scores compared to no music at 2 min post puncture. Black line: median (50th percentile), blue line: interquartile range (25%–75%), dashed line: Whiskers (1.5* interquartile range).

When including all patients (with and without baseline pain) only a slight tendence to lower VAS scores was observed for group 1 (Mozart) compared to group 4 (control group) 2 min after puncture (ANOVA 1: *p* = 0.179). However, when analyzing only patients with baseline pain, compared to the control group (group 4) the ANOVA 2 analysis showed significantly lower VAS scores for the patients listening to Mozart (group1) 2 min after puncture (*p* = 0.018) with borderline values 30 s after puncture (*p* = 0.054). No significant differences were observed for the other comparison of groups.

## 4 Discussion

To our knowledge, this is the first study evaluating the impact and time dependence over 2 min of different music types, including favorite music and Mozart, on pain perception during intravenous cannulation prior to MRI in adult patients with and without baseline pain.

The VAS was selected as the primary measure of pain as it offers several psychometric advantages relevant to our study design (41), has been shown to highly correlate with other validated scales, and has proven a high reliability and concurrent validity in repeated measurement within a short time interval (43, 44).

To some extent our results are akin to previous reports suggesting that most people would prefer to listen to favorite music during a painful procedure or in specific conditions, followed by a smaller trend selecting classical music (10, 30, 33). In our study, the pain reduction effect by favorite music was independent on the presence of baseline pain.

The observation that Mozart exerted a significant pain reduction effect 2 min and a borderline pain reduction effect 30 s after puncture when only patients with baseline pain were included suggests that the “Mozart effect” was driven by the patients manifesting baseline pain. Inversely, the only slight tendency towards pain reduction by Mozart in the analysis including all patients was mainly attributed to the patients without baseline pain. These findings infer that in terms of pain the Mozart effect may depend on whether baseline pain is present or not. However, our exploratory findings have to be interpreted with caution at this stage and require confirmation in future randomized studies including more patients and in which also pain of a higher intensity is applied.

Our findings also indicate a music-related time-dependence effect with the strength of pain reduction positively correlating with increasing time from venous puncture, as reflected by the strongest pain reduction at the latest time point measured, i.e., 2 min after puncture. This exploratory observation also needs to be validated in further studies. The causes may be a combination of factors (45, 46) including neurophysiological mechanisms (activation of emotional processes via the limbic system and prefrontal networks as well as endogenous pain inhibition which require some minutes after pain onset to be effective), physiological factors (time for adaption to music as attention may initially focus on the cannulation, time required for cognitive distraction and expectation perception upon music listening), and methodical aspects (dominance of the needle discomfort immediately after cannulation possibly suppressing the music effect). This kind of “distraction” by the needle (cannulation) should not be confused with the previously reported “task distraction” during music listening which has been shown to increase pain tolerance (47).

It would be informative to evaluate how far beyond 2 min our observed music-related time-dependence effect on pain may extend in future studies to better assess the therapeutical potential of music in painful situations.

The lacking effect of “no music” compared to Mozart and favorite music could allude to a positive effect of music for pain reduction in general. However, the lacking pain reduction impact by relaxation music indicates that music in general does not minimize pain perception *per se*, but that the extent of pain tolerance rather depends on the music genre played.

It could be argued that the patients without baseline pain may bias the results, since their scores could not decrease to minus values in the subsequent VAS assessment. However, Farrar et al. showed that the relationship between percent change in pain reduction and its representation in several pain-rating scales was consistent regardless of baseline pain (48). To prevent this bias, the baseline VAS scores of all patients were normalized. Further, our study involved an uneasy or painful procedure (venous puncture) and most of the patients without baseline pain experienced pain immediately after venous puncture (59/133, 2nd VAS assessment).

The patients in our study were exposed to a special condition with an imminent MRI requiring the body to be positioned in a tight space with the head fixed in a coil for 20–60 min. Most patients were suffering from clinical symptoms or already had been diagnosed a disease. Together with the additional uncertainty of the upcoming MRI results an uncomfortable situation linked to some sort of stress or anxiety (3) negatively influencing the patients’ well-being and psychological sureness could be expected, independently of the presence of pain. In case of baseline pain, a situation like this may even aggravate the pain. Therefore, it is very likely that the significant pain reduction by listening to favorite music, in intensity followed by Mozart, was not just mediated by a mere reduction in pain, e.g., by endorphin release triggering (45). Possibly, other factors played a role as well, e.g., physiological factors via a downregulation of the autonomic nervous system influencing blood pressure and heart rate as well as a reduction in anxiety and psychic stress leading to relaxation (1, 4, 49, 50) mediated by a down-regulation of the hypothalamus–pituitary–adrenal axis with subsequent lowering of cortisol concentration (51). The lacking relaxing or positive effect may be considered paradoxical for relaxation music, which however could be explained by a more difficult accessibility to relaxation music by individuals not familiar with it. Our chosen work of relaxation music was of rather constant low volume and very monotonous, possibly completely disregarded by the patients. In contrast, the work by Mozart was rather cheerful and much more dynamic with a faster tempo, probably being more effective in terms of distraction from pain and the exposed situation. The distraction effect may also be applied to favorite music, which is familiar to the individual, and easy and inviting to follow.

One might think that patients with a higher BMI may perceive less pain upon externally applied pain due to the subcutaneous fat tissue acting as a buffer. However, when analyzing the BMI as a possible variable on pain, no statistical influence was found, being in line with the available literature showing inconsistent results to this effect (52). Our results rather corroborate the positive effect of selected music in stressful or uneasy conditions independently of body weight (10).

This study has some limitations. First, we only included Mozart's KV 448 as a classical music piece, which hampers a general statement of the efficacy of classical music for pain relief. However, inclusion of more music works would have required a higher number of patients for a reliable statistical analysis. The intervention was rather small consisting in the application of an only light punctual pain with a rather small needle. The “patient—music group assignment” allocation rather represents a pseudo-randomization and not a true randomization, thus the results have to be treated with caution. Further, the subgroup of patient with baseline pain was rather small limiting the evidence of the exploratory findings. Future studies are necessary to evaluate the effect of music also on stronger pain application. The technicians in the MRI-room accompanying and talking to the patient could be regarded as a confounder as the mere presence of a person confidently talking to one may be perceived as calming and could distract the patient from the present situation and alter pain sensitivity. However, as all four groups were exposed to the same condition, this patient-technician interaction can—to a certain extent—be nullified across all groups.

As all technicians were similarly exposed to music while performing the venous puncture, the influence of music on the completion of the venous puncture was considered negligible.

## 5 Conclusion

We propose the inclusion of music for minor painful procedures in uneasy situations, such as intravenous cannulation for an MRI. Especially patients with baseline pain may benefit from listening to music. Patients more sensitive to stress or claustrophobia could also profit from music, which may reduce additional measures, such as the administration of relaxing substances. In this context, favorite music should be considered as most effective. Listening to Mozart's Sonata KV 448 may be considered as an alternative to favorite music in patients with baseline pain, as a sort of “Mozart effect” was only observed in this subgroup of patients. However, the results are exploratory and even if a trend of music effect was observed in painful situations, the extent of the effect by listening to Mozart in this context has still to be validated in future randomized studies.

## Data Availability

The raw data supporting the conclusions of this article will be made available by the authors, without undue reservation.
